# Dyadic Coping Across the Lifespan: A Comparison Between Younger and Middle-Aged Couples With Breast Cancer

**DOI:** 10.3389/fpsyg.2019.00404

**Published:** 2019-03-19

**Authors:** Chiara Acquati, Karen Kayser

**Affiliations:** ^1^Graduate College of Social Work, University of Houston, Houston, TX, United States; ^2^Renato LaRocca Chair of Oncology Social Work, Kent School of Social Work, University of Louisville, Louisville, KY, United States

**Keywords:** dyadic coping, couples, breast cancer, lifespan, mutuality

## Abstract

The association between dyadic coping and adjustment to cancer has been well-established. However, a significant gap in the literature is the understanding of how the life stage of couples may influence their dyadic coping and the accompanying quality of life. Although younger couples have been identified at higher risk for poor coping because of less collaborative behaviors and higher vulnerability to stress, only a limited number of studies have addressed younger women's coping with breast cancer in the context of close relationships. The present study addressed the differential impact of the illness on the quality of life and dyadic coping behaviors of younger and middle-aged dyads and the influence of relational mutuality on couples' coping in the two groups. A sample of 86 couples participated in a cross-sectional study; 35 younger couples were compared to 51 middle-aged dyads. Patients and partners completed measures of quality of life, dyadic coping, and mutuality. Independent-samples *t*-tests were used to examine differences in the two groups, while the Actor-Partner Interdependence Model (APIM) identified actor and partner effects of relational mutuality on dyadic coping. Younger women and their partners reported statistically significant worse quality of life and dyadic coping scores than the middle-age group. For younger couples, positive and negative coping styles were the result of both actor and partner effects of mutuality. The study highlighted the more negative impact of breast cancer on the quality of life of younger patients and partners. It also revealed a stronger influence of each partner's relational mutuality compared to the middle-age group in predicting both adaptive and maladaptive coping behavior. Future studies should continue to examine the developmental trajectory of dyadic coping across the lifespan in order to develop psychosocial interventions to promote younger dyads' coping efforts.

## Introduction

In the last 20 years a new attention toward interpersonal aspects of coping has emerged (Revenson et al., 2005; Kayser and Scott, 2008; Saita, 2009; Donato, 2012; Iafrate and Donato, 2012; Regan et al., 2015; Traa et al., 2015). This emphasis has resulted in the convergence of theoretical frameworks of close relationships and stress and coping with the goal to examine how coping develops within the context of significant relationships (Revenson et al., 2005; Iafrate and Donato, 2012). As a consequence, couples' coping has started to be conceptualized no longer referring to the separate perspectives of the two partners, but as a dyadic process involving their mutual influence (Bodenmann, 1997). Dyadic coping is conceptualized as a process shaped by the individual's close relationships (Bodenmann, 2005; Revenson et al., 2005; Peterson and Bush, 2013). It is described as “the interplay between the stress signals of one partner and the coping reactions of the other, a genuine act of shared coping” (Revenson et al., 2005; p. 4). Through a series of interactions, dyadic coping contributes to a sense of *we-ness* and promotes the conjoint creation of strategies to respond to the stressful event (Revenson, 1994; Bodenmann, 1997; Scott et al., 2004; Kayser et al., 2007).

The significance of dyadic coping for relationship functioning, psychological, and physical well-being has been established across several types of stressors (Bodenmann et al., 2004, 2010; Hinnen et al., 2008a,b; Randall and Bodenmann, 2009; Badr et al., 2010; Sullivan et al., 2010; Vilchinsky et al., 2010). Longitudinal studies have confirmed that dyadic coping represents a protective factor for the couple's relationship, with better relational outcomes and a reduced risk of being divorced registered among dyads reporting common coping (Bodenmann and Cina, 2005; Bodenmann et al., 2006). More recently, it has been established that dyadic coping significantly predicts relationship satisfaction and that aggregated positive forms of coping are stronger predictors of this outcome than negative ones (Falconier et al., 2015). In the context of cancer, the ability of the couple to face the illness as a unit contributes to higher relationship quality (Picard et al., 2005; Fergus and Gray, 2009; Badr et al., 2010; Traa et al., 2015). Similarly, better relationship functioning and quality of life are reported by couples that engage in relationship maintenance behaviors, social support exchanges, mutual constructive communication, and joint dyadic coping (Lavery and Clarke, 1999; Norton and Manne, 2007; Badr and Taylor, 2008; Langer et al., 2009; Hagedoorn et al., 2011a,b; Pasipanodya et al., 2012; Manne et al., 2015). However, a significant gap in the literature is the understanding of how the life stage of couples may influence their dyadic coping and the accompanying quality of life.

Previous research indicates that younger couples are more vulnerable to distress, experience poorer quality of life, and appear to be at risk for negative adjustment to the stress of the cancer because of four reasons. First, it has been documented that coping abilities increase with age, with older individuals presenting better emotion regulation and more effective collaborative coping skills (Folkman et al., 1987; Aldwin, 1994; Diehl et al., 1996; Labouvie-Vief, 2003; Revenson, 2003; Helgeson et al., 2004; Revenson and Pranikoff, 2005). Second, contextual and generational factors may impact younger couples' vulnerabilities, as younger generations report higher levels of stress, often associated with financial insecurity and un-healthy lifestyles, when compared with previous generations (American Psychological Association, 2015). Third, while there is little disagreement that marital satisfaction decreases with time (Bradbury and Karney, 2014), the literature about close relationships has provided over the last decades evidence of relational and psychological difficulties for newlyweds and younger families (Carstensen et al., 2003, 2011; Amato and Hohmann-Marriott, 2007; Luong et al., 2011; van Steenbergen et al., 2011; Scabini and Rossi, 2012; Woszidlo and Segrin, 2013; Bradbury and Karney, 2014). Researchers have recently identified different trajectories within the first years of marriage which are associated to marital dissolution. The most significant reduction in marital satisfaction is reported among couples who had low satisfaction at baseline (Lavner et al., 2012) or greatest expectations about the quality of their relationships (Lavner et al., 2013). Increased rates of divorce after 4 and 10 years of marriage have been associated to personality traits, stress, aggression, and poor communicative behaviors (Lavner and Bradbury, 2010). Investigations of the effects of personality traits and stressful events on marital satisfaction in recently married couples confirmed that partners' occupation, work interference, and family stress were negatively associated with marital satisfaction (van Steenbergen et al., 2011; Woszidlo and Segrin, 2013). Furthermore, economic hardship has been associated with higher rate of conflict (Halliday Hardie and Lucas, 2010), lower life satisfaction, higher pessimism (Haid and Seiffge-Krenke, 2012) and negative communication (Williamson et al., 2013) for newly-weds. In contrast, a study by Neff and Broady (2011) highlighted that adaptation to moderately stressful events early in the marriage is associated to reduced stress spillover effect, greater self-efficacy, and marital adjustment; suggesting that practicing stress adaptation strategies in the early years of marriage can lead to increased ability to cope with stress at a later stage of the couple's life. Finally, when dyadic coping has been investigated among different age groups and cohorts, authors have highlighted the complexity of the relational exchange within the dyad. While initial studies showed that older couples perform consistently better than younger ones (Revenson, 2003; Revenson and Pranikoff, 2005; Berg and Upchurch, 2007; Blanchard-Fields and Coats, 2008; Hoppmann et al., 2008), others have highlighted stereotypical similarity in younger couples (Iafrate et al., 2012). The authors concluded that younger partners appear to experience a heightened perceived idealization of the relationship. A recent study analyzed stress, dyadic coping, and partners' well-being in three age cohorts (Cohort 1: 20-35y.o.; Cohort 2: 40-55y.o.; Cohort 3 65-80 y.o.). Among the younger cohort, women's well-being was affected by stress and negative supportive behaviors, while males' quality of life was mostly influenced by individual coping and contextual factors (Vedes et al., 2015). Among middle-aged couples, the well-being of both partners was influenced by stress and dyadic coping. While investigators found an actor effect for female partners, the well-being of male partners appeared to be more dependent on the dyadic coping of the wife. These differences disappeared in the late-adulthood group. It is therefore possible to conclude that the relationship among stress, dyadic coping, and well-being changes across the life course experience.

If we extend our reflection on developmental differences to the experience of breast cancer, young women present higher psychological distress and poorer quality of life than older women (Hartl et al., 2010; Luutonen et al., 2011; So et al., 2011; Cataldo et al., 2013; Hau et al., 2013; Champion et al., 2014; Bantema-Joppe et al., 2015). Young women are also more vulnerable to the disruptions of the disease on their close and intimate relationships, reporting higher concerns for their relationship with partners, difficulties disclosing the diagnosis, and higher feelings of isolation (Ruddy et al., 2013; Ahmad et al., 2015). Anxiety and depressive symptoms have been found among those who had received chemotherapy and reported low level of support from close ones and partners (Borstelmann et al., 2015; Gold et al., 2015). The literature suggests that not all couples cope effectively with the stress of cancer. In a large prospective cohort study of women diagnosed with breast cancer at age 40 or younger, ~20% perceived the partner as unsupportive. For them, an increased likelihood to report anxiety symptoms existed (Borstelmann et al., 2015). Similarly, Avis et al. (2004) found that young women with higher levels of marital problems reported lower global, physical, emotional, and breast cancer-specific quality of life. This finding extends to survivorship, as younger survivors perceive less intimate or partner support than the older group, more social constraints, and lower marital satisfaction (Stava et al., 2006; Champion et al., 2014). For those experiencing difficulties in their relationship, reduced perceived benefit from the cancer experience and higher negative impact on their well-being and quality of life have been documented (Champion et al., 2014). Finally, Walsh et al. (2005) identified that although most women experienced greater closeness with their partners, 1 in 4 participants reported increased relational strain which ended in separation or end of the relationship 12% of the time. The illness also affects the quality of life of male partners (Baucom et al., 2005; Antoine et al., 2012; Duggleby et al., 2014; Hasson-Ohayon et al., 2014; Fergus et al., 2015; Borstelmann et al., 2017). Antoine et al. (2012) found that partners tended to be very close and supportive at the beginning of the cancer experience, providing high levels of mutual support, while over time they wished for the couple to resume a sense of normalcy. Other studies have highlighted (Vanlemmens et al., 2012a,b) the negative impact of the disease on psychological, physical, relational, social, sexual, domestic, professional, and economic dimensions. While couple cohesion assumed a central role for patients, caregiving concerns, and apprehension for the future became more relevant for younger partners (Christophe et al., 2015a,b). These findings are in line with a recent study conducted by Borstelmann et al. (2017) where partners of young breast cancer patients presented more maladaptive coping styles.

The present work addresses a significant gap in the literature about younger women with breast cancer by considering the impact of the diagnosis on both patients and their partners from a relational perspective. The study focuses on the concept of dyadic coping, therefore highlighting not only the impact of the illness on health-related quality of life, but extending our understanding of how young couples cope together with the illness and whether the processes are different from those of middle-aged dyads. In the present work, dyadic coping is conceptualized as a process of communicating stress and appraising coping behaviors that occur between partners (Systemic-Transactional Model; Bodenmann, 2005; Bodenmann et al., 2016). Relational mutuality, namely, the ability to be empathic with one's partner and to participate in a shared emotional experience (Jordan et al., 1991; Jordan, 2009), has emerged as an important antecedent for the enactment of coping behaviors (Kayser et al., 2007). Mutuality was found to be significantly associated to women's adjustment to cancer (Kayser et al., 1999) and it is a key relational quality that informs the coping strategies enacted by the partners according to the Relational-Cultural Model of Dyadic Coping (Kayser et al., 2007, 2010). Since we were interested in understanding how younger and middle-aged couples differ in the process of coping, the variable was included in our hypotheses and analysis.

The overall purpose of the present study was to examine differences between younger and middle-aged couples in terms of quality of life, dyadic coping, and mutuality and the influence of relational mutuality on couple's coping in the two groups. The following research questions guided this work:

1. Do younger breast cancer patients and partners differ from middle-aged patients and partners on their quality of life, dyadic coping, and relational mutuality?
  *Hypothesis 1.1:* Younger patients with breast cancer will report lower quality of life, relational mutuality, and higher negative dyadic coping styles compared to middle-aged breast cancer patients.
  *Hypothesis 1.2:* Younger partners will report lower quality of life, relational mutuality, and higher negative dyadic coping styles compared to middle-aged partners of breast cancer patients.
2. How does relational mutuality affect dyadic coping styles of breast cancer patients and their partners by age group?
  *Hypothesis 2.1*: Patients' perceived relational mutuality will influence their own dyadic coping style and their partners' dyadic coping style.
  *Hypothesis 2.2*: Partners' perceived relational mutuality will influence their own dyadic coping style and the patients' dyadic coping style.
  *Hypothesis 2.3*: Differences in actor and partner effects of relational mutuality on dyadic coping will exist by age group, between younger and middle-aged dyads.

## Materials and Methods

### Participants and Procedure

Participants were recruited among adult patients newly diagnosed with early-stage non-metastatic breast cancer in two medical centers in the northeast United States.^1^ Inclusion criteria were: (a) having received a diagnosis of primary non-metastatic breast cancer within the last 3 months; (b) being currently involved in a close relationship with a partner; (c) being older than 18 years of age; (d) receiving routine clinical care at the participating sites; and (e) being able to understand English. The study was inclusive of heterosexual and same-sex relationships. However, only one same-sex couple participated. Patients meeting the inclusion criteria were approached by a recruitment coordinator and questionnaires were mailed to those interested. The patients were sent letters at their home address, which included a study brochure, a decline card, and pre-stamped return envelopes. If feasible, the recruiters met with the woman and her partner in the clinic to present the study. Both the patient and partners/spouses needed to consent to be enrolled. Couples who agreed to be in the study were mailed survey questionnaires and returned their questionnaires in two separate pre-stamped envelopes. Ninety-four patients and ninety partners returned their questionnaires to the research team, with data available for 86 dyads.

In this study younger couples were identified as those where the patient was ≤45 years at diagnosis. The literature about breast cancer usually refers to “younger women” as those in their reproductive years (Hulvat and Jeruss, 2009). The mean age of menopause is at 51 years for American women with a peri-menopause stage between 47 and 51 years (National Institute of Aging, 2015). In addition, when survivorship has been investigated in a younger group of breast cancer patients, researchers have usually enrolled women from the age of 50, suggesting that they would have been ~45 years old at time of diagnosis (i.e., Champion et al., 2014). A total of 35 dyads met this criterion, and they were compared with the remaining 51 “middle-aged couples” composed by women who were between the ages of 46 and 66, with only one participant being 72. The mean age for middle-aged cancer patients was 55 (see Table 1).

**Table 1:** Socio-demographic, relational, and clinical characteristics of the sample.

**Variable**	**Younger couples**			**Middle-aged couples**		
	**Patients (*N* = 35)**	**Partners (*N* = 35)**	***p*-value**	**Patients (*N* = 51)**	**Partners (*N* = 51)**	***p*-value**
**AGE**			n.s.			<0.05
(mean score)	38.31 (SD = 4.78)	40.6 (SD = 6.65)		55.00 (SD = 5.74)	57.65 (SD = 6.97)	
**LENGTH OF RELATIONSHIP**			n.s.			n.s.
(mean score)	10.71 (SD = 5.75)			25.66 (SD = 11.64)		
**MARITAL STATUS**						n.s.
Married	32 (91.4%)			46 (90.2%)		
Not married	3 (8.6%)			5 (9.8%)		
**RACE**			n.s.			n.s.
Non-hispanic white	35 (100%)	32 (91.4%)		49 (96.1%)	47 (92.2%)	
Black	–	–		–	1 (2.0%)	
Asian	–	–		–	1 (2.0%)	
Latino	–	–		1 (2.0%)	–	
Native American/Indian				–	1 (2.0%)	
Unknown/Other	–	3 (8.65)		1 (2.0%)	1 (2.0%)	
**NUMBER OF CHILDREN**			n.s.			n.s.
0	8 (22.9%)	9 (25.7%)		8 (15.75)	4 (7.8%)	
1	7 (20.0%)	6 (17.15)		4 (7.8%)	5 (9.8%)	
2	10 (28.6%)	10 (28.6%)		21 (41.2%)	22 (43.1%)	
3	8 (22.9%)	8 (22.9%)		12 (23.5%)	14 (27.5%)	
4	2 (5.7%)	2 (5.7%)		5 (9.8%)	5 (9.8%)	
5	–	–		1 (2.0%)	1 (2.0%)	
**EDUCATION^a^**			n.s.			n.s.
Less than high school	–	2 (5.7%)		–	–	
High school graduate	1 (2.9%)	2 (5.7%)		4 (7.8%)	2 (3.9%)	
High school with some	4 (11.4%)	6 (17.1%)		6 (11.6%)	10 (19.6%)	
College	14 (40.0%)	10 (28.6%)		13 (25.5%)	11 (21.6%)	
College graduate	3 (8.6%)	7 (20%)		8 (15.7%)	4 (7.8%)	
College with some graduate	9 (25.7%)	5 (14.3%)		18 (35.3%)	15 (29.4%)	
Hours	4 (11.4%)	2 (5.7%)		2 (3.9%)	9 (17.6%)	
Master's degree Ph.D., MD, JD other	–	1 (2.9%)		–	–	
**OTHER^b^**			n.s.			n.s.
Unskilled labor	–	–		1 (2.0%)	–	
Managerial	5 (14.3%)	6 (17.1%)		3 (5.9%)	16 (31.4%)	
Homemaker/Parent	5 (14.3%)	–		7 (13.7%)	–	
Skilled labor	2 (5.7%)	5 (14.3%)		1 (2.0%)	2 (3.9%)	
Professional	21 (60.0%)	21 (60.0%)		32 (62.7%)	27 (52.9%)	
Other	2 (5.7%)	3 (8.6%)		7 (13.7%)	6 (11.8%)	
**INCOME**			n.s.			n.s.
≤ $10,000	–	–		1 (2.0%)	–	
$10,000−29,900	1 (2.9%)	1 (2.9%)		1 (2.0%)	1 (2.0%)	
$30,000−49,900	2 (5.7%)	1 (2.9%)		7 (13.7%)	5 (9.85)	
$50,000−69,900	6 (17.1%)	6 (17.1%)		10 (19.6%)	6 (11.8%)	
$70,000−89,900	8 (22.9%)	7 (20.0%)		1 (2.0%)	4 (7.8%)	
≥ $90,000	18 (51.4%)	20 (57.1%)		31 (60.8%)	35 (68.6%)	
**RELIGIOUS AFFILIATION**			n.s.			n.s.
Catholic	17 (48.6%)	15 (42.9%)		19 (37.3%)	15 (29.4%)	
Protestant	8 (22.9%)	7 (20.0%)		17 (33.3%)	20 (39.2%)	
Jewish	3 (8.6%)	3 (8.6%)		7 (13.7%)	10 (19.6%)	
Atheist/Agnostic	1 (2.9%)	5 (14.3%)		3 (5.9%)	4 (7.8%)	
Other	6 (17.1%)	5 (14.3%)		5 (9.8%)	2 (3.9%)	
**CURRENT MEDICATIONS**						
Yes	20 (57.1%)			32 (64.0%)		
No	15 (42.9%)			18 (36.0%)		
**CHEMOTHERAPY**						
Yes	10 (28.6%)			8 (16.35)		
No	25 (71.4%)			41 (83.7%)		
**PREVIOUS TREATMENT FOR DEPRESSION**			n.s.			n.s.
Yes	6 (17.1%)	7 (20.0%)		16 (31.4%)	10 (19.6%)	
No	29 (82.9%)	28 (80.0%)		35 (68.6%)	40 (78.4%)	
**TIMING OF TREATMENT DEPRESSION**						
Before cancer diagnosis	6 (17.1%)			14 (28.6%)		
After cancer diagnosis	–			2 (4.15)		
Before and after	–			1 (2.05)		
Not applicable	29 (82.9%)			32 (62.7%)		

a *Non-significant differences are detected also when the variable is recoded in 2 categories, 1 = High School, and 2 = College graduate*.

b *Non-significant differences are detected also when the variable is recoded in 2 categories. Unskilled labor, Homemaker, and other were recoded as 1, managerial, skilled labor and professional were recoded as 2. The Fisher's Exact Test indicates a 2-sided significance of 0.31*.

### Relational-Cultural Model of Dyadic Coping

The theoretical framework of the study derives from the Relational-Cultural Model of Dyadic Coping (Kayser et al., 2007) which proposes that appraisal and responses to cancer are shaped by relational characteristics; one of these characteristics is relational mutuality. The model is influenced by the Relational-Cultural Theory, a perspective that explains the individual sense of self as being in relation (Miller, 1984; Jordan et al., 1991; Jordan, 2009) and identifies the goal of human development in the acquisition of relational competence, which can be achieved by engaging in growth-fostering relationships. According to this conceptualization of dyadic coping, relationship awareness, authenticity, and mutuality determine the pattern of coping couples develop. Relationship awareness refers to the partners' awareness that the stressor is affecting both of them (Kayser et al., 2007; Kayser and Scott, 2008). Authenticity describes partners' ability to disclose genuine feelings to each other in a sensitive and appropriate way (Kayser et al., 2007; Kayser and Scott, 2008; Scott and Kayser, 2009). Finally, mutuality is defined as the ability to be empathic with the partner and to participate in a shared emotional experience (Jordan, 1997a,b; Feldman and Broussard, 2005, 2006; Kayser et al., 2007; Kayser and Scott, 2008). Depending on the presence of these characteristics, two different patterns of relational coping are enacted: mutual responsiveness or disengaged avoidance (Kayser et al., 2007; Kayser and Scott, 2008). Mutually responsive couples appraise the stress as affecting both members of the dyad, respond to each other's emotional and physical needs when coping, and grow through the stressful experience. On the contrary, disengaged avoidant couples will appraise the cancer as an individual stressor, avoid or deny the stress, and do not acknowledge benefits of the stress on their relationship (Kayser et al., 2007, 2010; Kayser and Scott, 2008). While the link between mutuality and dyadic coping can be bi-directional, based on the Relational-Cultural Theory, we hypothesize in this study that mutuality is a relational quality that is likely to influence the behaviors of positive dyadic coping.

### Measures

#### Quality of Life

The quality of life of women diagnosed with breast cancer was measured by the Functional Assessment of Cancer Therapy-Breast (FACT-B) Scale (Cella et al., 1993; Brady et al., 1997). The FACT-B (Version 4) is a 37-item measure that contains four general subscales assessing the physical, social/family, emotional, and functional well-being of the individual, along with the breast cancer-specific subscale that assesses concerns of particular relevance to breast cancer patients (e.g., body image, arm swelling and tenderness). Patients were invited to indicate how true each statement has been for them in the previous seven days, and items are rated on a 5 point Likert scale ranging from “Not at All” (0) to “Very Much” (4). The FACT-B consists of five subscale scores: physical well-being (PWB), social/family well-being (SWB), emotional well-being (EWB), functional well-being (FWB) and additional concerns (BCS), with higher scores indicating higher quality of life. From these subscale scores, two assessment total scores were calculated: the FACT-B total score, and the FACT-G score. The FACT-B total score is calculated by summing all five un-weighted subscale scores, with total scores in the range of 0–136. The FACT-G score is calculated by summing PWB, SWB, EWB, and FWB scores, with scores in the range of 0–108. Administration and scoring guidelines are available on the website http://www.facit.org/FACITOrg. The FACT-B has been extensively used in psychosocial oncology research and has demonstrated high validity and internal consistency (Cella et al., 1993; Brady et al., 1997; Winstead-Fry and Schultz, 1997; Webster et al., 1999, 2003; Overcash et al., 2001). In its validation study, Cronbach's alpha for the total score was 0.90, with subscale alpha coefficients ranging from 0.63 to 0.86 (Brady et al., 1997). Evidence supported test-retest reliability, as well as convergent and divergent validity (Cella et al., 1993; Brady et al., 1997; Winstead-Fry and Schultz, 1997; Webster et al., 1999, 2003; Overcash et al., 2001). For the purpose of the present investigation, the five subscales and the FACT-G and FACT-B total scores were used when comparing quality of life between the two groups of cancer patients. Similarly to the data available in the literature, high internal consistency has been registered (FACT-G α = 0.90, FACT-B total score α = 0.90, PWB α = 0.88, SWB = 0.81, EWB α = 0.83, FWB α = 0.85, BCS α = 0.81).

Due to the unavailability of a measure of quality of life for both cancer patients and partners at time of the study, the Emotional Functioning subscale from the Quality of Life Questionnaire for Spouses (QL-SP) (Ebbesen et al., 1990) and the Illness Intrusiveness Ratings Scale (IIRS) (Binik et al., 1990) investigated quality of life among partners. The Emotional Function Dimension (14 items) examines the well-being of the individual in the previous 2 weeks by rating on a 7 point Likert scale anxiety, depression, concerns, frustration, and helplessness (Ebbesen et al., 1990). Total scores range from 7 to 98, with higher scores indicating better functioning. The scale demonstrated high internal consistency in previous studies (Feldman and Broussard, 2006; Iafrate et al., 2012), and Cronbach's alpha was 0.91 in the present sample. The Illness Intrusiveness Ratings Scale measures the interference of the partner's illness and treatment on 13 dimensions (Devins et al., 1983; Devins, 1994) on a 7 point Likert Scale. The total score ranges from 13 to 91, with higher scores indicating greater impact of the patient's illness on the partner. Several studies support the reliability and validity of the instrument (Binik et al., 1990; Devins et al., 1990; Devins, 1994). A systematic review highlighted that Cronbach's alpha scores ranged from the 0.80's to the 0.90's across studies (Devins, 2010). In the present sample the Cronbach's alpha was 0.88.

#### Dyadic Coping

Dyadic coping was measured by the Dyadic Coping Scale (Bodenmann, 2000). This self-report questionnaire assesses stress communication and dyadic coping as perceived by each partner, each partner's perception of the other's coping, and each partner's view of how they cope as a couple. In this version each item (for a total of 61) is measured on a 6-point Likert scale. The Dyadic Coping Scale contains five subscales: Stress Communication (SC), Common (CDC), Positive (PDC), Hostile (HDC) and Avoidance of Dyadic Coping (ADC). Stress communication refers to the partners' ability to communicate emotion- and problem-focused stress. Examples are “I ask my partner to do things for me when I have too much to do,” “I try to hide my stress from my partner so that he/she does not notice it,” and “I tell my partner openly how I feel and that I would appreciate his/her support.” Common dyadic coping occurs when both members of the couple experience the stressful event and they participate in the coping process in a symmetric or complementary way. They use strategies like joint problem solving, information seeking, and mutual commitment. Examples of items of this subscale are “We are supportive of each other and help one another out,” “We help one another to put the problem in perspective and see it in a new light,” and “We caress one another and make love.” Positive dyadic coping refers to the use of supportive dyadic coping strategies like the provision of practical help, information, advice, and understanding and helping to relieve tension. Examples of items included in this subscale are: “My partner gives me the feeling that he/she understands me”; “My partner listens to me and gives me the opportunity to communicate the entire situation,” and “My partner takes on things that I normally do in order to help me out.” Hostile dyadic coping indicates a situation when the stress signals of one partner originate a hostile behavior by the other. Responses or behaviors that can be considered hostile include distancing, ridicule, sarcasm, clear disinterest and minimizing the emotional experience of stress of the other. Scale items that are included in this subscale are: “I make fun of my partner's stress,” “I let my partner know that I do not want to be bothered with his/her problems,” and “Although my partner makes time for me, his/her thoughts are somewhere else.” Finally, avoidance of dyadic coping describes ambivalent and superficial coping responses, where authentic engagement is absent (Bodenmann, 1997, 2005). Examples are “When my partner is stressed, I tend to get out of his/her way,” and “When my partner is stressed I tend to withdraw.” Satisfactory psychometrics of the questionnaire have been reported (Feldman and Broussard, 2006). In this study, reliability scores ranged from 0.68 to 0.96 for patients, and from 0.68 to 0.95 for partners. Cronbach's alpha of each subscale for patients and partners is hereby reported for each subscale: SC: 0.68 patients and partners; CDC: 0.86 patients, 0.83 partners, PDC: 0.96 patients; 0.95 partners; HDC: 0.80 patients; 0.70 partners; ADC: 0.68 patients, 0.68 partners. The Cronbach's alpha for the total score were 0.91 patients and 0.90 partners.

#### Relational Mutuality

Mutuality is defined as a “bidirectional expression of feelings, thoughts, and activity between individuals in a relationships” (Genero et al., 1992. p. 36) and involves elements of empathy, engagement, authenticity, zest, diversity and empowerment (Miller, 1986). It can be summarized as the ability to experience feelings of another person, while maintaining a sense of one's own feelings, therefore being authentic and able to empower the other member of the dyad. We used the 22-item Mutual Psychological Development Questionnaire (MPDQ) (Genero et al., 1992) that was developed to measure mutuality in adult close relationships. The MPDQ contains items from two relationship perspectives—the self and other. It consists of items assessing the six dimensions of mutuality mentioned above. Examples of items include: “When we talk about things that matter to my spouse/partner, I am likely to be receptive/ get impatient /try to understand” and “When we talk about things that matter to me, my spouse/partner is likely to pick up on my feelings/ feel like we are not getting anywhere, share similar experiences.” Hence, scores are summed in order to compute the level of mutuality reported by each person when considering the close relationship in exam, and for this reason we use the term relational mutuality in the present work. In the present sample reliability scores were high for both patients (α = 0.93) and partners (α = 0.91).

### Data Analysis

The analysis was conducted in accordance with the recommendations of the University of Louisville Institutional Review Board. The present work was approved through the expedited review procedure because it involved materials that have been already collected and because it involved no more than minimal risk. After obtaining IRB approval, IBM SPSS Statistics 22 was used for data screening and data analysis. Descriptive statistics were obtained and mean substitution was implemented to handle missing data on the key variables. A Missing Value Analysis (MVA) was conducted on all the variables included in the dataset and revealed that missing data ranged from 0.6 to 2.3% of cases on variables of interest (from 1 to a max of 4 cases). Pearson *r* correlations were used to assess the linear relationship between socio-demographic, clinical, and psychosocial measures. Comparisons of demographic characteristics between patients and spousal caregivers were calculated using paired sample *t*-tests for continuous variables and chi-square for ordinal and categorical variables. Differences between younger and middle-aged breast cancer patients and their partners on quality of life, dyadic coping, and mutuality have been assessed by calculating independent samples *t*-test. Then, the Actor-Partner Interdependence Model (APIM) was used to examine actor and partner effects of relational mutuality on the dyadic coping style of each member of the dyad in the two separate groups (Kenny et al., 2006).

## Results

### Sample Description

Descriptive statistics for the demographic, relational, and clinical characteristics of the two groups are presented in Table 1. Younger patients were on average in their late thirties (*M* = 38.31, *SD* = 4.78). The majority of them were college educated and working as professionals and reported an income higher than $90,000 per year. Their partners were on average 40 years old (*SD* = 6.65). They were highly educated and could be considered to be middle to upper-middle class. The average length of the relationship was of 11 years (*SD* = 5.75), with most couples being married (91.4%).

Middle-aged couples had been in a relationship for ~26 years (*SD* = 11.6) and most of them were married (90.2%). Patients were in their mid-fifties (*M* = 55, *SD* = 5.74), were highly educated, and working in professional settings (62.7%). Only 16.3 % of middle-age women were currently receiving chemotherapy. Partners were in their late fifties (*M* = 57, *SD* = 6.97), highly educated, and could be categorized as middle to upper-middle class. There were differences between patients and partners for age and occupation, with partners being significantly older (*t*_(100)_) = −2.09, *p* < 0.05) and in more managerial and professional positions (Table 1).

In terms of the variables of interest, younger women and their partners reported similar scores on common, hostile, and avoidance of dyadic coping. However, younger women reported higher scores than their partners on stress communication (*p* < 0.001) and positive dyadic coping behaviors (*p* < 0.05). Younger women reported affected quality of life in all the subscales of the FACT-B and partners showed levels of moderately affected emotional well-being and illness intrusiveness. Middle-aged dyads were characterized by elevated scores on dyadic coping styles like positive and common dyadic coping; indicating that the two partners were utilizing both individual and relational resources to cope with the cancer diagnosis. Middle-aged couples had low scores on hostile and avoidance dyadic coping. Women had elevated scores on the subscales that address physical, social, and overall quality of life. The areas mostly affected by the cancer diagnosis appeared to be their emotional and functional well-being. Middle-aged partners presented high scores on relational mutuality, emotional well-being, and low intrusiveness (Tables 2 and 3).

**Table 2:** Descriptives of the major study variables for younger couples.

	**Patients**		**Partners**			
**Variables**	***M***	**SD**	***M***	**SD**	***t***	***p***
**DYADIC COPING**						
Stress communication	4.14	0.79	3.37	0.70	4.36	<0.001^***^
Common dyadic coping	3.53	0.67	3.51	0.52	0.15	0.88
Positive dyadic coping	4.15	0.75	3.75	0.56	2.58	<0.05^*^
Hostile dyadic coping	2.07	0.58	2.13	0.45	−0.46	0.64
Avoidance of dyadic coping	2.79	0.89	2.69	0.75	0.50	0.61
Relational mutuality	4.38	0.70	4.45	0.48	−0.53	0.59
**QUALITY OF LIFE PATIENTS**						
Physical well-being	18.51	6.25				
Social well-being	21.52	4.07				
Emotional well-being	14.28	4.98				
Functional well-being	17.60	5.65				
Breast cancer symptoms	22.58	5.70				
FACT-G	72.36	15.50				
FACT-B	95.03	19.35				
**QUALITY OF LIFE PARTNERS**						
Emotional well-being			58.83	12.91		
Illness intrusiveness			43.06	14.12		

* p < 0.05,

^**^p < 0.01,

*** *p < 0.001*.

**Table 3:** Descriptives of the major study variables for Middle-Aged Couples.

	**Patients**		**Partners**			
**Variables**	***M***	**SD**	***M***	**SD**	***t***	***p***
**DYADIC COPING**						
Stress communication	4.26	0.67	3.49	0.76	5.37	<0.001^***^
Common dyadic coping	3.65	0.79	3.63	0.69	0.13	0.89
Positive dyadic coping	4.15	0.85	3.94	0.74	1.32	0.18
Hostile dyadic coping	1.90	0.49	1.80	0.47	1.00	0.19
Avoidance of dyadic coping	2.61	0.85	2.51	0.68	0.63	0.52
Relational mutuality	4.46	0.63	4.56	0.55	−0.87	0.39
**QUALITY OF LIFE PATIENTS**						
Physical well-being	22.17	4.73				
Social well-being	23.27	4.48				
Emotional well-being	17.51	2.73				
Functional well-being	19.30	4.97				
Breast cancer symptoms	25.66	4.26				
FACT-G	82.21	11.75				
FACT-B	107.75	13.46				
**QUALITY OF LIFE PARTNERS**						
Emotional well-being			68.47	12.58		
Illness intrusiveness			31.70	13.56		

^*^p < 0.05,

^**^p < 0.01,

*** *p < 0.001*.

### Differences Between Younger and Middle-Aged Patients and Partners on Quality of Life, Relational Mutuality, and Dyadic Coping

Independent-samples *t*-tests compared the mean scores of quality of life, relational mutuality, and dyadic coping styles of younger and middle-aged breast cancer patients and partners. Significant differences between younger and middle-age breast cancer patients were identified for physical (*p* < 0.01), emotional well-being (*p* < 0.01), and impact of breast cancer symptoms (*p* < 0.01). Younger women in this sample indicated higher physical and emotional difficulties, additional concerns related to breast cancer symptoms such as body appearance, social support and interaction. These results were also confirmed when the total scores of the scale were considered. No differences existed between the two groups for relational mutuality and dyadic coping style (Table 4).

**Table 4:** Independent samples *t-*test comparing dyadic coping, relational mutuality, and quality of life among Younger and Middle-Age breast cancer patients.

**Variable**	**Age group**	***M***	**SD**	***T***	***p***
Stress communication	Younger patients	4.14	0.79	−0.71	0.48
	Middle-age patients	4.26	0.67		
Common dyadic coping	Younger patients	3.53	0.67	−0.77	0.44
	Middle-age patients	3.65	0.79		
Positive dyadic coping	Younger patients	4.15	0.75	0.04	0.96
	Middle-age patients	4.15	0.85		
Hostile dyadic coping	Younger patients	2.07	0.57	1.45	0.15
	Middle-age patients	1.90	0.49		
Avoidance of dyadic coping	Younger patients	2.79	0.89	0.94	0.35
	Middle-age patients	2.61	0.85		
Relational Mutuality	Younger patients	4.38	0.70	−0.57	0.57
	Middle-age patients	4.46	0.63		
Physical well-being	Younger patients	18.51	6.25	−2.94	0.005^**^
	Middle-age patients	22.17	4.73		
Social well-being	Younger patients	21.51	4.06	−1.84	0.06
	Middle-age patients	23.27	4.48		
Emotional well-being	Younger patients	14.28	4.98	−3.48	0.001^**^
	Middle-age patients	17.50	2.73		
Functional well-being	Younger patients	17.60	5.65	−1.47	0.14
	Middle-age patients	19.30	4.97		
Breast cancer symptoms	Younger patients	22.59	5.69	−2.86	0.005^**^
	Middle-age patients	25.67	4.26		
FACT-G	Younger patients	72.36	15.50	−3.12	0.001^**^
	Middle-age patients	82.21	11.75		
FACT-B	Younger patients	95.03	19.35	−3.17	0.002^**^
	Middle-age patients	107.75	13.46		

^*^p < 0.05,

** p < 0.01,

^***^p < 0.001.

When the two groups of partners were compared on the variables of interest, the results of the independent samples *t*-test indicated that the younger group scored higher on maladaptive dyadic coping styles, presented lower mean scores of stress communication, common, and positive dyadic coping, and worse quality of life than the middle-aged partners (Table 5). Significant differences were found between the two groups of partners for hostile dyadic coping (*p* < 0.01), emotional well-being (*p* < 0.01) and illness intrusiveness (*p* < 0.001). From the present analysis it is possible to affirm that younger partners experienced the illness to be more intrusive in their life, had worse emotional well-being, and were more likely to perceive their dyadic coping as characterized by disinterest or minimizing the seriousness of the partner's stress than those reported by middle-age partners.

**Table 5:** Independent samples *t-*test comparing dyadic coping, relational mutuality, and quality of life among Younger and Middle-Age partners.

**Variable**	**Age group**	***M***	**SD**	***t***	***p***
Stress communication	Younger partners	3.37	0.70	−0.75	0.45
	Middle-age partners	3.49	0.77		
Common dyadic coping	Younger partners	3.51	0.52	−0.93	0.33
	Middle-age partners	3.63	0.69		
Positive dyadic coping	Younger partners	3.75	0.54	−1.41	0.18
	Middle-age partners	3.94	0.71		
Hostile dyadic coping	Younger partners	2.13	0.45	3.16	0.002^**^
	Middle-age partners	1.80	0.47		
Avoidance of dyadic coping	Younger partners	2.69	0.75	1.14	0.25
	Middle-age partners	2.51	0.68		
Relational mutuality	Younger partners	4.45	0.48	−0.97	0.33
	Middle-age partners	4.56	0.55		
Emotional well-being	Younger partners	58.83	12.91	−3.45	0.001^**^
	Middle-age partners	68.47	12.58		
Illness intrusiveness	Younger partners	43.06	14.12	3.75	<0.001^***^
	Middle-age partners	31.71	13.56		

^*^p < 0.05,

** p < 0.01,

*** *p < 0.001*.

### Actor and Partner Effects of Relational Mutuality on Dyadic Coping in Younger and Middle-Aged Couples

Patients and partners' mean-centered scores on relational mutuality were regressed on the outcome variable in a single regression model to examine whether self-reported levels of relational mutuality of patients and partners predicted the individual's engagement in different dyadic coping styles, by conducting separate APIM analyses in the two groups for each style of coping. To facilitate a clear understanding of the analysis and avoid confusion with the partner effect, partners will be identified with the term “caregivers” in the present paragraph. Prior to the analysis, the actor and partner scores were grand-mean centered and the variable role was coded as 1 for patients, and −1 for caregivers. An Intraclass Correlation Coefficient was calculated to assess non-independence between the scores of the partners. Then, an Omnibus Test of Distinguishability was conducted to assess whether treating the dyad as distinguishable improved the fit of the model. We then tested whether role acted as a moderator of actor and/or partner effects. Hence, an interaction model using REML estimation was tested first, followed by a two-intercept approach. Power estimates were obtained using G^*^Power 3.1.9.2., and the results of the power analysis were favorable. Results indicate an average effect size of 0.164 for the younger group and 0.118 for the middle-aged group, with a sample of 35 and 51 dyads, *p*-value of 0.05, and 0.80 power.

#### Stress Communication

In both groups of couples there is evidence of an actor effect of relational mutuality on stress communication (*p* < 0.001 for younger couples; *p* < 0.05 for middle age dyads) Individuals reporting high levels of mutuality were more likely to engage in stress communication behaviors. From our results there was no evidence that having a partner who reported higher mutuality was associated with the person's use of this coping strategy. Furthermore, mean level differences were predicted for stress communication of patients and informal caregivers, with younger and middle-age patients reporting more frequent use of this coping strategy than their respective partners/spouses.

#### Common Dyadic Coping

**Figure 1:**
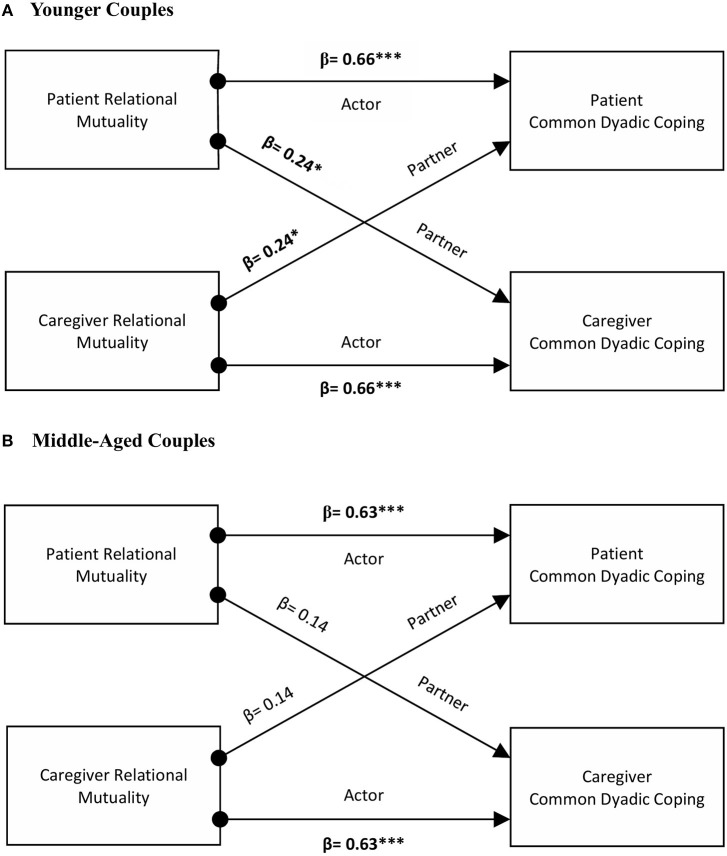
The actor and partner effects of relational mutuality as predictors of common dyadic coping in younger and middle-aged couples. ^*^p < 0.05, ^**^p < 0.01, ^***^p < 0.001.

Among younger dyads, the analysis revealed the presence of actor and partner effects of relational mutuality on common dyadic coping. No significant interaction of role by actor or partner effects occurred. Younger patients and caregivers reporting high levels of mutuality were more likely to engage in common dyadic coping behaviors (β = 0.66, *p* < 0.001). There was also evidence that having a partner who scores high on mutuality was associated with the person's use of common coping strategies (β = 0.24, *p* < 0.05). On the contrary, among middle-aged dyads an actor effect was found (β = 0.63, *p* < 0.001), indicating that high scores on mutuality were associated with an increase in their own dyadic coping score. The interaction role by actor effect approached significance (*p* = 0.055) (see Figure 1).

#### Positive Dyadic Coping

For younger dyads, an actor effect of relational mutuality on positive dyadic coping was identified (β = 0.79, *p* < 0.001). Younger individuals reporting high levels of mutuality were more likely to engage in positive dyadic coping behaviors. Furthermore, among younger couples, significant role differences were found, with patients predicted to report higher scores (*p* < 0.01). Among couples in the middle-age group there was evidence of an actor effect (*p* < 0.001) and that gender was a significant moderator of the actor effect for women and caregivers (*p* < 0.05). To examine the actor by role interaction for men and women separately, simple slopes were calculated. Results indicated that both patients and caregivers actor effects were significant and that the actor effect was greater for patients (patients: β = 0.82, *p* < 0.001; caregivers: β = 0.48, *p* < 0.001).

#### Hostile Dyadic Coping

**Figure 2:**
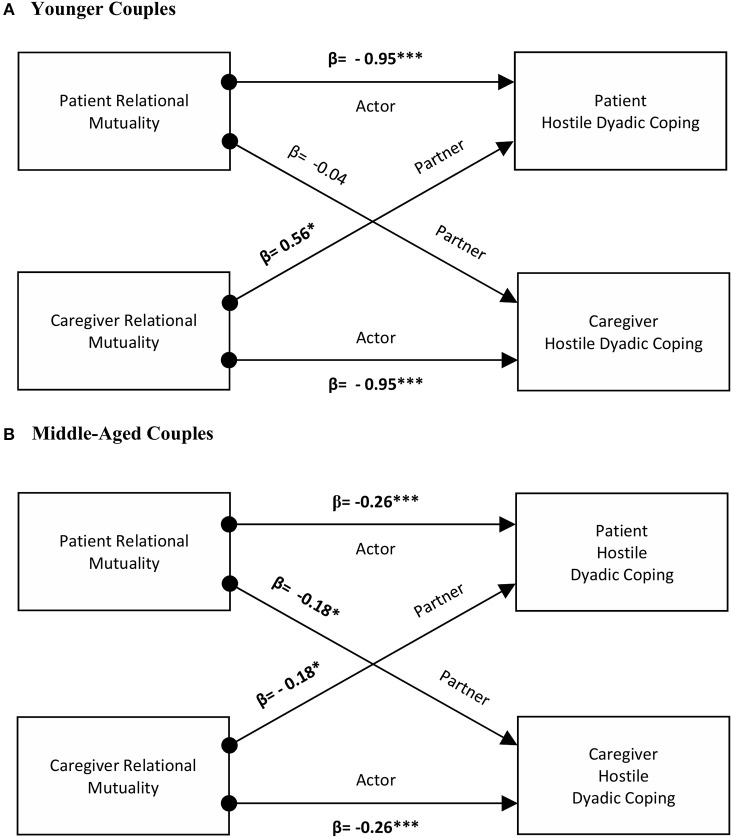
The actor and partner effects of relational mutuality as predictors of hostile dyadic coping in younger and middle-aged couples. ^*^p < 0.05, ^**^p < 0.01, ^***^p < 0.001.

Actor and partner effects were reported for the younger couples, with significant interactions of role by partner effects. Younger individuals reporting high levels of relational mutuality were less likely to report hostile dyadic coping (β = −0.95, *p* < 0.001). To test whether the partner effect differed by patients and caregivers, simple slopes were calculated. The caregiver partner effect was not significant (*p* = 0.70). In contrast, for patients, higher mutuality scores of the caregiver were associated with an increase in hostile dyadic coping scores (β = 0.56, *p* < 0.05). The interaction role by actor effect approached significance (*p* = 0.06). Among middle-aged dyads evidence existed for both an actor and partner effects. Patients and caregivers presenting higher levels of mutuality were more likely to report lower levels of hostile dyadic coping (β = −0.26, *p* < 0.001). Similarly, having a partner reporting high scores on mutuality was associated with reduced hostile coping (β = −0.18, *p* < 0.05). No evidence for differences of actor and partner effects by role was identified in this group of couples (see Figure 2).

#### Avoidance of Dyadic Coping

Among younger couples, an actor effect was detected (β = −0.72, *p* < 0.001). Present findings indicate that as relational mutuality increases, this was associated with reduced scores on avoidance of dyadic coping in younger participants, with no interaction of actor effect by role. For middle-aged couples both actor (β = −0.32, *p* < 0.001) and partner effects (β = −0.020, *p* < 0.05) of relational mutuality were identified. Higher self-reported scores on mutuality were associated with reduced avoidance of dyadic coping. Similarly, having a partner scoring high on mutuality was associated to lower scores on this coping style.

## Discussion

In the last decade there has been an increasing recognition that younger women with breast cancer represent a separate group among all women diagnosed with the disease because of unique clinical and psychosocial issues. Among the most relevant problems reported by younger women, there is an increased concern for their relationship with the partner. However, despite the evidence that has identified more difficulties and challenges for young couples, a limited number of contributions has investigated the experience of younger women in the context of their close relationships. The present study examined younger couples' coping with breast cancer by comparing them to a group of middle-aged dyads. Results of this study confirm the differential impact of the illness on quality of life and coping responses of younger and middle-aged couples. Younger patients and their partners' adjustment to cancer appears to be significantly compromised within the first 3 months from diagnosis, with impaired functioning and worse quality of life. Younger women in this sample experienced elevated side effects of treatment and more difficult adjustment to the illness. These findings are consistent, despite the small sample size, with results from larger studies on the quality of life of younger women with breast cancer (Avis et al., 2004, 2005; Kroenke et al., 2004; Baucom et al., 2005; Luutonen et al., 2011; Howard-Anderson et al., 2012). In particular, when the same instrument (FACT-B) has been administered to younger patients, other authors have identified the presence of significant differences between younger and middle-aged breast cancer patients' overall quality of life (Avis et al., 2005; DiSipio et al., 2008; Brennan et al., 2014), with more negative physical well-being, emotional well-being, and breast cancer symptoms which are consistent with our results (Park et al., 2011; So et al., 2011).

Similarly, younger partners were more negatively impacted by the diagnosis and the illness in their quality of life, with statistically significant higher intrusiveness, lower emotional well-being, and a higher use of hostile dyadic coping compared to partners of middle-aged breast cancer patients. These results are similar to those of studies that found a more detrimental effect of the diagnosis on the quality of life of younger partners, who are faced with concerns about everyday life, negative affectivity, apprehension about the future and the couple relationship (Antoine et al., 2012; Vanlemmens et al., 2012a,b; Duggleby et al., 2014; Hasson-Ohayon et al., 2014; Christophe et al., 2015a,b; Fergus et al., 2015; Borstelmann et al., 2017). Finally, the higher score on illness intrusiveness is a finding that is consistent with the literature about cancer caregiving, which has identified higher burden, mood disturbance, and worse quality of life for those who assume this role at a younger age (Kim et al., 2012; Sjolander et al., 2012; Lee et al., 2013; Shahi et al., 2014).

The use of dyadic data analysis furthered our understanding of the role of relational mutuality among couples coping with breast cancer. Separate APIM models on younger and middle-aged dyads revealed the interaction between patients' and caregivers' mutuality scores and how they are associated to different coping behaviors. Among middle-aged dyads relational mutuality was associated with reduced hostile and avoidance of dyadic coping, suggesting that these couples present mutual emotional responsiveness and this ability contributes to reduced coping behaviors that may compromise the relationship. At the same time, the study presents evidence about the relational exchange that characterizes young couples facing cancer. In our sample, younger dyads presented elevated interdependence as evidenced by the fact that both adaptive and maladative dyadic coping strategies were the result of patients' and partners' perceived mutuality. The most interesting differences between the two groups pertain to common and hostile dyadic coping. Higher scores for common dyadic coping existed for the younger group as a consequence of actor and partner effects, suggesting that higher scores for this coping style are the result of the individual's self-reported scores, as well as the score of the partner. The results obtained for hostile dyadic coping and mutuality seem to suggest that younger couples may be vulnerable to situations where partners are not able to equally exchange thoughts, feelings, and actions (Jordan, 1997b). In contrast, among middle-aged couples, relational mutuality was associated to lower hostile dyadic coping for both. This finding for the younger group was unexpected and it can be potentially explained as a reverse causation due to the cross-sectional nature of the study. It is also possible to hypothesize that this result indicates the need for interventions aimed at promoting more beneficial relational exchanges in younger dyads and to enhance communication strategies that facilitate the beneficial disclosure of feelings. While it is not possible to elaborate more on this finding at this time, overall our results support the need for greater attention to the adjustment of couples facing cancer earlier in their relationship.

Some implications emerge from these findings. First, our results support the role of a relationship characteristic like relational mutuality in the enactment of dyadic coping behaviors, as it has been demonstrated in previous studies (Kayser, 2005; Kayser et al., 2007, 2010; Kayser and Scott, 2008). This result echoes the literature that identifies relationship characteristics as antecedents of dyadic coping (Staff et al., 2017): stability (Bodenmann and Cina, 2005), satisfaction (Bodenmann et al., 2008; Berg et al., 2011; Landis et al., 2014; Ruffieux et al., 2014), and quality of the relationship (Bodenmann, 1997; Wise et al., 2010; Bergstraesser et al., 2015) have been found to promote coordinated forms of coping with stressors (Staff et al., 2017). Second, our study highlighted how, despite no significant differences were found between the dyadic coping scores of younger and middle-aged patients and partners (with the exception of Hostile Dyadic Coping in partners), dyadic coping responses were associated with different patterns of reciprocal influence among the two members of the dyad. Both positive and negative dyadic coping behaviors in the younger group resulted from the scores of the partners on relational mutuality. While the role of the two partners' mutuality may be more salient for younger dyads, the ability to communicate effectively their mutual empathic responsiveness may be limited in this group, while middle-aged couples implement more coordinated patterns of communication. This consideration is supported by studies where couples in longer relationships were better able to use dyadic coping styles than partners in shorter relationships (Wunderer and Schneewind, 2008; Papp and Witt, 2010; Herzberg, 2013; Staff et al., 2017). This differential ability may be also the result of developmental processes across the lifespan (Berg and Upchurch, 2007). Better competence in the ability to regulate emotions and appraisal of stress is also reported in older individuals, which tend to show greater mutuality and less maladaptive coping (Folkman et al., 1987; Aldwin, 1994; Diehl et al., 1996; Labouvie-Vief, 2003). The second developmental aspect is the temporal process of dyadic coping. Studies have highlighted the changing nature of dyadic coping over time, especially in the case of an illness (Fang et al., 2001; Martire et al., 2002; Helgeson et al., 2004; Schulz and Schwarzer, 2004). Again, age-related differences have been identified because younger individuals report greater distress and reduced ability to perform collaborative coping (Helgeson et al., 2004; Revenson and Pranikoff, 2005). In contrast, older adults become better able to cope effectively (Revenson, 2003). These findings support the need to develop interventions aimed at promoting not only the couples' ability to cope together with the illness, but also to enhance communication strategies that facilitate the beneficial disclosure of feelings, thoughts, and emotions between the two partners.

The study presents several limitations, such as small sample size, homogeneous sample composition, and cross-sectional design. The sample size affects the generalization of results to other groups, and it was not possible to elaborate on differences between participants and non-participants and potential selection biases. A more properly powered sample is needed to investigate the differential impact of breast cancer among patients and partners across the developmental trajectory. Although difficulties in the recruitment of couples for research are well-established by the literature (Kenny et al., 2006; Fredman et al., 2009; Regan et al., 2013; Hagedoorn et al., 2015), the limited sample and the use of different instruments to measure quality of life among partners impacted the possibility to test more complex models of dyadic data analysis. Furthermore, in the current study the identification of “younger” and “middle-aged” dyads was derived only by the age of the patient at diagnosis. Future studies can benefit from a more in-depth exploration of the role of developmental stages, duration of relationship, and cohort effects (Baucom et al., 2012; Revenson and Lepore, 2012). The sample was largely homogeneous in regards to race, socio-economic status, sexual orientation, and education. This project used a cross-sectional design; hence, it is not possible to elaborate whether the difference between the younger and middle-aged couples' coping persist over time. A newer version of the instrument measuring dyadic coping has been introduced (Dyadic Coping Inventory, Bodenmann, 2008) and it has been validated for the use in the US population as this study was completed (Levesque et al., 2014a; Randall et al., 2016). The inclusion of this new instrument in future research projects is recommended.

## Implications for Practice

Findings from the present study suggest the need for a renewed attention to the psychosocial issues of patients and partners (Institute of Medicine, 2008, 2013). Psychosocial providers and healthcare professionals need to develop greater understanding of how to work effectively with younger patients and their significant ones, and to promote their ability to find support within the health care system. The identification of unique needs and stressors requires the utilization of screening and assessment strategies able to capture the life experience of younger women and their well-being, in order to promote effective and timely referrals. Screening and assessment should be inclusive of the significant other in order to identify individuals who may have difficulties coping with the patient's diagnosis and its demands.

Since younger patients' and their partners' relational mutuality was associated to dyadic coping, psychosocial interventions should address the dyad as a unit of intervention. Over the last two decades, several couple-based interventions have been developed and tested in RCTs (Baik and Adams, 2011; Regan et al., 2012; Badr and Krebs, 2013). Despite these efforts, more investigation is warranted to evaluate their effectiveness and application in practice settings. Programs aimed at supporting younger dyads coping with breast cancer should assist participants identifying their relationships' qualities, positive and negative coping patterns, and their impact on both individuals' quality of life. Through this experience younger couples should be enabled to communicate effectively with each other and to establish new coping repertoires (Skerrett and Fergus, 2015). It is, however, necessary to adapt existing protocols to target topics that are relevant for couples in the early years of their relationship, marriage or for cohabitating couples (Ponzetti, 2016). Additional topics to address as part of these interventions could include: social relations with families of origin and the extensive supportive network, financial planning, intimacy and sexual functioning, fertility preservation, transition to parenthood or strategies to cope with cancer-related infertility. While preliminary data indicate beneficial changes in communication, closeness, and relationship strengths (Fergus et al., 2014, 2015), it will be important to further investigate factors associated with positive results, timing of the intervention, and the mechanism for therapeutic change (Revenson and DeLongis, 2011; Revenson and Lepore, 2012).

## Conclusion

The present study contributes to the understanding of the experience of younger couples coping with breast cancer. This work has highlighted the more negative effect of the illness on the quality of life of the two partners. From this investigation it emerges that patients and partners' relational mutuality scores influence the dyadic coping strategies enacted to respond to the stress of the illness. Among younger couples it appears that both positive and maladaptive outcomes in terms of couple's coping are the results of patients' and partners' mutuality. It follows that both members of the dyad have an essential role in the development of coping strategies that will promote better adjustment to the disease and the preservation of their relationship. Hence, these findings contribute to the current theoretical reflection about the process of dyadic coping and its association to individual and relational outcomes (Levesque et al., 2014b; Regan et al., 2015; Traa et al., 2015; Staff et al., 2017). Future studies, both cross-sectional and longitudinal, are needed to examine the differential impact cancer has on couples across the developmental trajectory and to provide confirmation to these results. Building on larger samples, this research will lead to better understand sources of stress and relational difficulties experienced by younger dyads, which will be critical in developing couple-based interventions to promote their coordinated coping efforts.

## Author Contributions

CA contributed to the study design, statistical analysis, and to the writing of the manuscript's introduction, materials and methods, results, discussion, and references sections. KK contributed to the study design, data collection, and to the writing of the introduction, results and discussion sections. All authors reviewed and approved manuscript for publication.

### Conflict of Interest Statement

The authors declare that the research was conducted in absence of any commercial or financial relationships that could be construed as a potential conflict of interest.

## Footnotes

^1^This study was part of a larger intervention study and included only baseline data collected before couples were randomly assigned to the treatment arms (see Kayser et al., 2010 for further information).

## References

[B1] Ahmad S. Fergus K. McCarthy M. (2015). Psychosocial issues experienced by young women with breast cancer: the minority group with the majority of need. Curr. Opin. Support Palliat. Care 9, 271–278. 10.1097/SPC.0000000000000162 26147915

[B2] Aldwin C. M. (1994). Stress, Coping, and Development: An Integrative Perspective. New York, NY: Guilford.

[B3] Amato P. R. Hohmann-Marriott B. (2007). A comparison of high- and low-distress marriages that end in divorce. J. Marriage Fam. 69, 621–638. 10.1111/j.1741-3737.2007.00396.x

[B4] American Psychological Association (2015). Stress in America. Paying With Our health. Available online at: https://www.apa.org/news/press/releases/stress/2014/stress-report.pdf

[B5] Antoine P. Vanlemmens L. Fournier E. Trocme M. Christophe V. (2012). Young couples' experiences of breast cancer during hormone therapy. a phenomenological interpretative dyadic analysis. Cancer Nurs. 36, 213–220. 10.1097/NCC.0b013e31826429a5 22964867

[B6] Avis N. E. Crawford S. Manuel J. (2004). Psychosocial problems among younger women with breast cancer. Psychooncology 13, 295–308. 10.1002/pon.744 15133771

[B7] Avis N. E. Crawford S. Manuel J. (2005). Quality of life among younger women with breast cancer. J. Clin. Oncol. 23, 3322–3330. 10.1200/JCO.2005.05.130 15908646

[B8] Badr H. Carmack C. L. Kashy D. A. Cristofanilli M. Revenson T.A. (2010). Dyadic coping in metastatic breast cancer. Health Psychol. 29, 169–180. 10.1037/a0018165 20230090 PMC3138118

[B9] Badr H. Krebs P. (2013). A systematic review and meta-analysis of psychosocial interventions for couples coping with cancer. Psychooncology 22, 1688–1704. 10.1002/pon.3200 23045191 PMC3562417

[B10] Badr H. Taylor L. C. (2008). Effects of relationship maintenance on psychological distress and dyadic adjustment among couples coping with lung cancer. Health Psychol. 27, 616–627. 10.1037/0278-6133.27.5.616 18823188 PMC9976549

[B11] Baik O. M. Adams K. B. (2011). Improving the well-being of couples facing cancer: a review of couples based psychosocial interventions. J. Marital Fam. Ther. 37, 250–266. 10.1111/j.1752-0606.2010.00217.x 21457288

[B12] Bantema-Joppe E. J. de Bock G. H. Woltman-van Iersel M. Busz D. M. Ranchor A. V. Langendijk J. A.. (2015). The impact of age on changes in quality of life among breast cancer survivors treated with breast-conserving surgery and radiotherapy. Br. J. Cancer 112, 636–643. 10.1038/bjc.2014.632 25602967 PMC4333491

[B13] Baucom D. H. Kirby J. S. Pukay-Martin N. D. Porter L. S. Fredman S. J. Gremore T. M.. (2012). Men's psychological functioning in the context of women's breast cancer. J. Marital Fam. Ther. 38, 317–329. 10.1111/j.1752-0606.2009.00133.x 22512294

[B14] Baucom D. H. Porter L. S. Kirby J. S. Gremore T. M. Keefe F. J. (2005). Psychosocial issues confronting young women with breast cancer. Breast Dis. 23, 103–113. 10.3233/BD-2006-23114 16823173

[B15] Berg C. A. Upchurch R. (2007). A developmental-contextual model of couples coping with chronic illness across the adult life span. Psychol. Bull. 133, 920–954. 10.1037/0033-2909.133.6.920 17967089

[B16] Berg C. A. Wiebe D. J. Butner J. (2011). Affect covariation in marital couples dealing with stressors surrounding prostate cancer. Gerontology 57, 167–172. 10.1159/000318642 20616529

[B17] Bergstraesser E. Inglin S. Hornung R. Landolt M. A. (2015). Dyadic coping of parents after the death of a child. Death Stud. 39, 128–138. 10.1080/07481187.2014.920434 25204680

[B18] Binik Y. M. Chowanec G. D. Devins G. M. (1990). Marital role strain, illness intrusiveness, and their impact on marital and individual adjustment in end-stage renal disease. Psychol. Health 4, 245–257. 10.1080/08870449008400394

[B19] Blanchard-Fields F. Coats A. H. (2008).The experience of anger and sadness in everyday problems impacts age differences in emotion regulation. Dev. Psychol. 44, 1547–1556. 10.1037/a0013915 18999321

[B20] Bodenmann G. (1997). Dyadic coping: a systemic-transactional view of stress and coping among couples: theory and empirical findings. Eur. Rev. Appl. Psychol. 47, 137–140.

[B21] Bodenmann G. (2000). Stress und Coping bei Paaren [Stress and Coping in Couples]. Göttingen: Hogrefe.

[B22] Bodenmann G. (2005). Dyadic coping and its significance for marital functioning, in Couples Coping with Stress: Emerging Perspectives on Dyadic Coping, eds. Revenson T. A. Kayser K. Bodenmann G. (Washington, DC: American Psychological Association), 33–49.

[B23] Bodenmann G. (2008). Dyadisches Coping Inventar (DCI) [Dyadic Coping Inventory]. Bern: Huber.

[B24] Bodenmann G. Atkins D. C. Schär M. Poffet V. (2010). The association between daily stress and sexual activity. J. Fam. Psychol. 24, 271–279. 10.1037/a0019365 20545400

[B25] Bodenmann G. Charvoz L. Widmer K. Bradbury T. N. (2004). Differences in individual and dyadic coping among low and high depressed, partially remitted, and nondepressed persons. J. Psychopathol. Behav. Assess. 26, 75–85. 10.1023/B:JOBA.0000013655.45146.47

[B26] Bodenmann G. Cina A. (2005). Stress and coping among stable-satisfied, stable-distressed and separated/divorced Swiss couples: a 5-years prospective longitudinal study. J. Divorce Remarriage 44, 71–89. 10.1300/J087v44n01_04

[B27] Bodenmann G. Pihet S. Kayser K. (2006). The relationship between dyadic coping and marital quality: a 2-year longitudinal study. J. Fam. Psychol. 20, 485–493. 10.1037/0893-3200.20.3.485 16938007

[B28] Bodenmann G. Plancherel B. Beach S. R. Widmer K. Gabriel B. Meuwly N.. (2008). Effects of coping-oriented couples therapy on depression: a randomized clinical trial. J. Consult. Clin. Psychol. 76, 944–954. 10.1037/a0013467 19045963

[B29] Bodenmann G. Randall A. K. Falconier M. K. (2016). Coping in couples: the Systemic transactional Model (STM), in Couples Coping With Stress: A Cross-Cultural Perspective, eds. Falconier M. K. Randall A. K. Bodenmann G. (New York, NY: Routledge), 2–22.

[B30] Borstelmann N. Shoshana M. Rosenberg S. M. Shari I. Gelber S. I. Meyer M. E. (2017). Partners of young breast cancer survivors: a cross-sectional evaluation of psychosocial issues and mental health. J. Clin. Oncol. 35 (Suppl. 5S):184 10.1200/JCO.2017.35.5_suppl.184 33000705

[B31] Borstelmann N. A. Rosenberg S. M. Ruddy K. J. Tamimi R. M. Gelber S. Schapira L.. (2015). Partner support and anxiety in young women with breast cancer. Psychooncology 24, 1679–1685. 10.1002/pon.3780 25765893

[B32] Bradbury T. N. Karney B. R. (2014). Intimate Relationships. New York, NY: WW Norton and Company.

[B33] Brady M. J. Cella D. F. Mo F. Bonomi A. E. Tulsky D. S. Lloyd S. R.. (1997). Reliability and validity of the functional assessment of cancer therapy-breast quality-of-life instrument. J. Clin. Oncol. 15, 974–986. 10.1200/JCO.1997.15.3.974 9060536

[B34] Brennan M. Gormally J. Butow P. Boyle F. Spillane A. (2014). Survivorship care plans in cancer: a systematic review of care plan outcomes. Br. J. Cancer 111, 1899–1908. 10.1038/bjc.2014.505 25314068 PMC4229639

[B35] Carstensen L. L. Fung H. Charles S. (2003). Socioemotional selectivity theory and the regulation of emotion in the second half of life. Motiv. Emot. 27, 103–123. 10.1023/A:1024569803230

[B36] Carstensen L. L. Turan B. Scheibe S. Ram N. Ersner-Hershfield H. Samanez-Larkin G. R.. (2011). Emotional experience improves with age: evidence based on over 10 years of experience sampling. Psychol. Aging 26, 21–33. 10.1037/a0021285 20973600 PMC3332527

[B37] Cataldo J. K. Paul S. Cooper B. Skerman H. Alexander K. Aouizerat B.. (2013). Differences in the symptom experience of older versus younger oncology outpatients: a cross-sectional study. BMC Cancer 13:6. 10.1186/1471-2407-13-6 23281602 PMC3576303

[B38] Cella D. F. Tulsky D. S. Gray G. Sarafian B. Lloyd S. Linn E. (1993). The Functional Assessment of Cancer Therapy (FACT) scale: development and validation of the general measure. J. Clin. Oncol. 11, 570–579. 10.1200/JCO.1993.11.3.570 8445433

[B39] Champion V. L. Wagner L. I. Monahan P. O. Daggy J. Smith L. Cohee A.. (2014). Comparison of younger and older breast cancer survivors and age-matched controls on specific and overall quality of life. Cancer 120, 2237–2246. 10.1002/cncr.28737 24891116 PMC4158315

[B40] Christophe V. Duprez C. Congard A. Antoine P. Lesur A. Fournier E.. (2015a). The subjective experience of young women with non-metastatic breast cancer: the young women with breast cancer inventory. Health Qual. Life Outcomes 13:73. 10.1186/s12955-015-0273-x 26036192 PMC4451721

[B41] Christophe V. Duprez C. Congard A. Fournier E. Lesur A. Antoine P.. (2015b). Evaluate the subjective experience of the disease and its treatment in partners of young women with non-metastatic breast cancer. Eur. J. Cancer Care 25, 734–743. 10.1111/ecc.12327 26013877

[B42] Devins G. M. (1994). Illness intrusiveness and the psychosocial impact of lifestyle disruptions in chronic life-threatening disease. Adv. Ren. Replace. Ther. 1, 251–263. 10.1016/S1073-4449(12)80007-0 7614328

[B43] Devins G. M. (2010). Using the illness intrusiveness ratings scale to understand health-related quality of life in chronic disease. J. Psychosom. Res. 68, 591–602. 10.1016/j.jpsychores.2009.05.006 20488277

[B44] Devins G. M. Binik Y. M. Hutchinson T. A. Hollomby D. J. Barré P. E. Guttmann R. D. (1983). The emotional impact of end-stage renal disease: importance of patients' perceptions of intrusiveness and control. Int. J. Psychiatry Med. 13, 327–343. 10.2190/5DCP-25BV-U1G9-9G7C 6671863

[B45] Devins G. M. Mandin H. Hons R. B. Burgess E. D. Klassen J. Taub K.. (1990). Illness intrusiveness and quality-of-life in end-stage renal disease: comparison and stability across treatment modalities. Health Psychol. 9, 117–142. 10.1037/0278-6133.9.2.117 2331973

[B46] Diehl M. Coyle N. Labouvie-Vief G. (1996). Age and sex differences in strategies of coping and defense across the life span. Psychol. Aging. 11, 127–139. 10.1037/0882-7974.11.1.127 8726378

[B47] DiSipio T. Hayes S. Newman B. Janda M. (2008). Health-related quality of life 18 months after breast cancer: comparison with the general population of Queensland, Australia. Support. Care Cancer. 16, 1141–1150. 10.1007/s00520-007-0392-y 18197429

[B48] Donato S. (2012). Il coping diadico, ovvero far fronte allo stress insieme: una rassegna della letteratura [dyadic coping, that is managing stress together: a review of the literature]. Giornale Italiano di Psicologia, 41, 473–504. 10.1421/78499

[B49] Duggleby W. Doell H. Cooper D. Thomas R. Ghosh S. (2014). The quality of life of male spouses of women with breast cancer: hope, self-efficacy, and perceptions of guilt. Cancer Nurs. 37, E28–E35. 10.1097/NCC.0b013e31827ca807 23348665

[B50] Ebbesen L. S. Guyatt G. H. MCCartney N. Oldridge N. B. (1990). Measuring quality of life in cardiac spouses. J. Clin. Epidemiol. 43, 481–487. 10.1016/0895-4356(90)90137-E 2324789

[B51] Falconier M. K. Jackson J. B. Hilpert P. Bodenmann G. (2015). Dyadic coping and relationship satisfaction: a meta-analysis. Clin. Psychol. Rev. 42, 28–46. 10.1016/j.cpr.2015.07.002 26295276

[B52] Fang C. Y. Manne S. L. Pape S. J. (2001). Functional impairment, marital quality, and patient psychological distress as predictors of psychological distress among cancer patients' spouses. Health Psychol. 20, 452–457. 10.1037/0278-6133.20.6.452 11714188

[B53] Feldman B. N. Broussard C. A. (2005). The influence of relational factors on men's adjustment to their partners' newly diagnosed breast cancer. J. Psych. Oncol. 23, 23–43. 10.1300/J077v23n02_03 16492650

[B54] Feldman B. N. Broussard C. A. (2006). Men's adjustment to their partners' breast cancer: a dyadic coping perspective. Health Soc. Work 31, 117–127. 10.1093/hsw/31.2.117 16776029

[B55] Fergus K. Ahmad S. McLeod D. L. Stephen J. Gardner S. Pereira A. (2015). Couplelinks- an online intervention for young women with breast cancer and their male partner: study protocol for a randomized controlled trial. Trials 16:33 10.1186/s13063-014-0534-8 25630357 PMC4336511

[B56] Fergus K. D. Gray R. E. (2009). Relationship vulnerabilities during breast cancer: patient and partner perspectives. Psychooncology 18, 1311–1322. 10.1002/pon.1555 19353517

[B57] Fergus K. D. McLeod D. Carter W. Warner E. Gardner S. Granek L.. (2014). Development and pilot testing of an online intervention to support young couples' coping and adjustment to breast cancer. Eur. J. Cancer Care 23, 481–492. 10.1111/ecc.12162 24472013

[B58] Folkman S. Lazarus R. S. Pimley S. Novacek J. (1987). Age differences in stress and coping processes. Psychol. Aging 2, 171–184. 10.1037/0882-7974.2.2.171 3268206

[B59] Fredman S. J. Baucom D. H. Gremore T. M. Castellani A. M. Kallman T. A. Porter L. S.. (2009). Quantifying the recruitment challenges with couple-based interventions for cancer: applications to early-stage breast cancer. Psychooncology 18,667–673. 10.1002/pon.1477 19061201 PMC4506748

[B60] Genero N. P. Baker Miller J. Surrey J. Baldwin L. M. (1992). Measuring perceived mutuality in close relationships: validation of the mutual psychological development questionnaire. J. Fam. Psychol. 6, 36–48. 10.1037/0893-3200.6.1.36

[B61] Gold M. Dunn L. B. Phoenix B. Paul S. M. Hamolsky D. Levine J. D.. (2015). Co-occurrence of anxiety and depressive symptoms following breast cancer surgery and its impact on quality of life. Eur. J. Oncol. Nurs. 20, 97–105. 10.1016/j.ejon.2015.06.003 26187660 PMC4706814

[B62] Hagedoorn M. Dagan M. Puterman E. Hoff C. Meijerink W. J. Delongis A.. (2011a). Relationship satisfaction in couples confronted with colorectal cancer: the interplay of past and current spousal support. J. Behav. Med. 34, 288–297. 10.1007/s10865-010-9311-7 21222025 PMC3141841

[B63] Hagedoorn M. Hein F. L. Schulz T. van der Heide J. J. H. Niesing J. Westerhuis R.. (2015). Are patient and relationship variables associated with participation of intimate partners in couples research? Health Psychol. 34, 270–273. 10.1037/hea0000141 25133832

[B64] Hagedoorn M. Puterman E. Sanderman R. Wiggers T. Baas P. C. van Haastert M.. (2011b). Is self-disclosure in couples coping with cancer associated improvement in depressive symptoms? Health Psychol. 30, 753–762. 10.1037/a0024374 21688913

[B65] Haid M. L. Seiffge-Krenke I. (2012). Effects of (un)employment on young couples' health and life satisfaction. Psychol. Health 28, 284–301. 10.1080/08870446.2012.720983 22963526

[B66] Halliday Hardie J. Lucas A. (2010). Economic factors and relationship quality among young couples: comparing cohabitation and marriage. J. Marriage Fam. 72, 1141–1154. 10.1111/j.1741-3737.2010.00755.x 21691414 PMC3116270

[B67] Hartl K. Schennach R. Muller M. Engel J. Reinecker H. Sommer H.. (2010). Quality of life, anxiety, and oncological factors: a follow-up study of breast cancer patients. Psychosomatics 51, 112–123. 10.1176/appi.psy.51.2.112 20332286

[B68] Hasson-Ohayon I. Goldzweig G. Dorfman C. Uziely B. (2014). Hope and social support utilisation among different age groups of women with breast cancer and their spouses. Psychol. Health 29, 1303–1319. 10.1080/08870446.2014.929686 24874894

[B69] Hau E. Browne L. Capp A. Delaney G. P. Fox C. Kearsley J. H.. (2013). The impact of breast cosmetic and functional outcomes on quality of life: long-term results from the St. George and Wollongong randomized breast boost trial. Breast Cancer Res. Treat. 139, 115–123. 10.1007/s10549-013-2508-z 23580069

[B70] Helgeson V. S. Snyder P. Seltman H. (2004). Psychological and physical adjustment to breast cancer over 4 years: identifying distinct trajectories of change. Health Psychol. 23, 3–15. 10.1037/0278-6133.23.1.3 14756598

[B71] Herzberg P. Y. (2013). Coping in relationships: the interplay between individual and dyadic coping and their effects on relationship satisfaction. Anxiety Stress Coping 26, 136–153. 10.1080/10615806.2012.655726 22300303

[B72] Hinnen C. Hagedoorn M. Ranchor A. V. Sanderman R. (2008a). Relationship satisfaction in women: a longitudinal case-control study abpout the role of breast cancer, personal assertiveness, and partners' relationship-focused coping. Br. J. Health Psychol. 13, 737–754. 10.1348/135910707X252431 17999780

[B73] Hinnen C. Ranchor A. V. Sanderman R. Snijders T. A. Hagedoorn M. Coyne J. C. (2008b). Course of distress in breast cancer patients, their partners, and matched control couples. Ann. Behav. Med. 36, 141–148. 10.1007/s12160-008-9061-8 18797979

[B74] Hoppmann C. A. Coats A. H. Blanchard-Fields F. (2008). Goals and everyday problem solving: examining the link between age-related goals and problem-solving strategy use. neuropsychology, development, and cognition. Neuropsychol. Dev. Cogn. B Aging Neuropsychol. Cogn. 15, 401–423. 10.1080/13825580701533777 17899456

[B75] Howard-Anderson J. Ganz P. A. Bower J. E. Stanton A. L. (2012). Quality of life, fertility concerns, and behavioral health outcomes in younger breast cancer survivors: a systematic review. J. Natl. Cancer Inst. 104, 386–405. 10.1093/jnci/djr541 22271773

[B76] Hulvat M. C. Jeruss J. S. (2009). Maintaining fertility in young women with breast cancer. Curr. Treat. Options Oncol. 10, 308–317. 10.1007/s11864-010-0116-2 20238254 PMC2908234

[B77] Iafrate R. Bertoni A. Donato S. Finkenauer C. (2012). Perceived similarity and understanding in dyadic coping among young and mature couples. Pers. Relatsh. 19, 401–419. 10.1111/j.1475-6811.2011.01369.x

[B78] Iafrate R. Donato S. (2012). Coping in a relational context: the case of dyadic coping, in Handbook of the Psychology of Coping: New Research, eds. Molinelli B. Grimaldo V. (Hauppauge, NY: Nova Science Publishers), 11–132.

[B79] Institute of Medicine (2008). Cancer Care for the Whole Patient: Meeting Psychosocial Health Needs. Washington, DC: The National Academies Press. 20669419

[B80] Institute of Medicine (2013). Delivering High Quality Cancer Care. Charting a New Course For a System in Crisis. Washington, DC: The National Academies Press. 24872984

[B81] Jordan J. (1997a). The relational model is a source of empowerment for women, in Women, Men, and Gender: Ongoing Debates, ed. Walsh M. R. (New Haven, CT: Yale), 373–382.

[B82] Jordan J. (1997b). Women's Growth in Diversity. New York, NY: Gilford Press.

[B83] Jordan J. V. Kaplan A. G. Miller J. B. Stiver I. P. Surrey J. L. (1991). Women's Growth in Connection: Writings from the Stone Center. New York, NY: Guilford Press.

[B84] Jordan V. J. (2009). Relational-Cultural Therapy. Washington, DC: American Psychological Association.

[B85] Kayser K. (2005). Enhancing dyadic coping during a time of crisis: a theory-based intervention with breast cacner patients and their partners, in Couples Coping with Stress, eds. Revenson T. A. Kayser K. Bodenmann G. (Washington, DC: American Psychological Association), 175–194.

[B86] Kayser K. Feldman B. N. Borstelmann N. A. Daniels A. A. (2010). Effects of a randomized couple-based intervention on quality of life of breast cancer patients and their partners. Soc. Work Res. 34, 20–32. 10.1093/swr/34.1.20

[B87] Kayser K. Scott J. L. (2008). Helping Couples Cope With Women's Cancers. An Evidence-Based Approach for Practitioners. New York, NY: Springer.

[B88] Kayser K. Sormanti M. Strainchamps E. (1999). Women coping with cancer: the influence of relationship factors on psychosocial adjustment. Psychol. Women Q 23, 725–739. 10.1111/j.1471-6402.1999.tb00394.x

[B89] Kayser K. Watson E. Andrade J. T. (2007). Cancer as a we-disease: examining the process of coping from a relational perspective. Fam. Syst. Health 25, 404–418. 10.1037/1091-7527.25.4.404

[B90] Kenny D. A. Kashy D. A. Cook W. L. (2006). Dyadic Data Analysis. New York, NY: The Guilford Press.

[B91] Kim Y. Spillers R. L. Hall D. L. (2012). Quality of life of family caregivers 5 years after a relative's cancer diagnosis: follow-up of the national quality of life survey for caregivers. Psychooncology 21, 273–281. 10.1002/pon.1888 22383269

[B92] Kroenke C. H. Rosner B. Chen W. Y. Kawachi I. Colditz G. A. Holmes M. D. (2004). Functional impact of breast cancer by age at diagnosis. J. Clin. Oncol. 22, 1849–1856. 10.1200/JCO.2004.04.173 15143077

[B93] Labouvie-Vief G. (2003). Dynamic integration: affect, cognition, and the self in adulthood. Curr. Dir. Psychol. Sci. 12, 201–206. 10.1046/j.0963-7214.2003.01262.x

[B94] Landis M. Bodenmann G. Bradbury T. N. Brandstätter V. Peter-Wight M. Backes S. (2014). Commitment and dyadic coping in long-term relationships. J. Gerontopsychol. Geriatr. Psychiatry 27, 139–149. 10.1024/1662-9647/a000112

[B95] Langer S. L. Brown J. D. Syrjala K. L. (2009). Intrapersonal and interpersonal consequences of protective buffering among cancer patients and caregivers. Cancer 115, 4311–4325. 10.1002/cncr.24586 19731352 PMC2762643

[B96] Lavery J. F. Clarke V. A. (1999). Prostate cancer: patients' and spouses' coping and marital adjustment. Psychol. Health Med. 4, 279–302. 10.1080/135485099106225

[B97] Lavner J. A. Bradbury T. N. (2010). Patterns of change in newlyweds' marital satisfaction. J. Marriage Fam. 72, 1171–1187. 10.1111/j.1741-3737.2010.00757.x 21116452 PMC2992446

[B98] Lavner J. A. Bradbury T. N. Karney B. R. (2012). Incremental change or initial differences? testing two models of marital deterioration. J. Fam. Psychol. 26, 606–616. 10.1037/a0029052 22709260 PMC3513382

[B99] Lavner J. A. Karney B. R. Bradbury T. N. (2013). Newlyweds' optimistic forecasts of their marriage: for better or for worse? J. Fam. Psychol. 27, 531–540. 10.1037/a0033423 23795607 PMC3758370

[B100] Lee Y. H. Liao Y. C. Liao W. Y. Shun S. C. Liu Y. C. Chan J. C.. (2013). Anxiety, depression and related factors in family caregivers of newly diagnosed lung cancer patients before first treatment. Psychooncology 22, 2617–2623. 10.1002/pon.3328 23893960

[B101] Levesque C. Lafointaine M. Caron A. Flesch J. L. Bjornson S. (2014b). Dyadic empathy, dyadic coping, and relationship satisfaction: a dyadic model. Eur. J. Psychol. 10, 118–134. 10.5964/ejop.v10i1.697

[B102] Levesque C. Lafontaine M.-F. Caron A. Fitzpatrick J. (2014a). Validation of the english version of the dyadic coping inventory. Meas. Eval. Couns. Dev. 47, 215–225. 10.1177/0748175614522272

[B103] Luong G. Charles S. T. Fingerman K. L. (2011). Better with age: Social relationships across adulthood. J. Soc. Pers. Relat. 28, 9–23. 10.1177/0265407510391362 22389547 PMC3291125

[B104] Luutonen S. Vahlberg T. Eloranta S. Hyvari H. Salminen E. (2011). Breast cancer patients receiving postoperative radiotherapy: Distress, depressive, symptoms and unmet needs of psychosocial support. Radiother. Oncol. 100, 299–303. 10.1016/j.radonc.2011.01.014 21316782

[B105] Manne S. L. Kissane D. Zaider T. Kashy D. Lee D. Heckman C.. (2015). Holding back, intimacy, and psychological and relationship outcomes among couples coping with prostate cancer. J. Fam. Psychol. 29, 708–719. 10.1037/fam0000096 26192132 PMC5225663

[B106] Martire L. M. Stephens M. A. P. Druley J. A. Wojno W. C. (2002). Negative reactions to received spousal care: predictors and consequences of miscarried support. Health Psychol. 21, 167–176. 10.1037/0278-6133.21.2.167 11950107

[B107] Miller J. B. (1984). The Development of Women's Sense of Self. Work in Progress No. 12. Wellesley, MA: Stone Center Working Papers Series.

[B108] Miller J. B. (1986). Toward a New Psychology of Women. Boston, MA: Beacon Press.

[B109] National Institute of Aging (2015). Health and Aging: Menopause. Available online at: https://www.nia.nih.gov/health/publication/menopause

[B110] Neff L. A. Broady E. F. (2011). Stress resilience in early marriage: can practice make perfect? J. Pers. Soc. Psychol. 101, 1050–1067. 10.1037/a0023809 21688919

[B111] Norton T. R. Manne S. L. (2007). Support concordance among couples coping with cancer: relationship, individual, and situational factors. J. Soc. Pers. Relat. 24, 675–692. 10.1177/0265407507081454

[B112] Overcash J. Extermann M. Parr J. Perry J. Balducci L. (2001). Validity and reliability of the FACT-G scale for use in the older person with cancer. Am. J. Clin. Oncol. 24, 591–596. 10.1097/00000421-200112000-00013 11801761

[B113] Papp L. M. Witt N. L. (2010). Romantic partners' individual coping strategies and dyadic coping: implications for relationship functioning. J. Fam. Psychol. 24, 551–559. 10.1037/a0020836 20954765 PMC3220915

[B114] Park B. Lee S. Lee A. R. Lee K. H. Hwang S. Y. (2011). Quality of life differences between younger and older breast cancer patients. J. Breast Cancer 14, 112–118. 10.4048/jbc.2011.14.2.112 21847405 PMC3148538

[B115] Pasipanodya E. C. Parrish B. P. Laurenceau J. P. Cohen L. H. Siegel S. D. Graber E. C.. (2012). Social constraints on disclosure predict daily well-being in couples coping with early-stage breast cancer. J. Fam. Psychol. 26, 661–667. 10.1037/a0028655 22686265

[B116] Peterson G. W. Bush K. R. (2013). Handbook of Marriage and the Family, 3rd Edn. New York, NY: Springer 10.1007/978-1-4614-3987-5

[B117] Picard L. Dumont S. Gagnon P. Lessard G. (2005). Coping strategies among couples adjusting to primary breast cancer. J. Psychosoc. Oncol. 23, 115–135. 10.1300/J077v23n02_08 16492655

[B118] Ponzetti J. J. Jr. (2016). Evidence-Based Approaches to Relationship and Marriage Education. New York, NY: Routledge.

[B119] Randall A. K. Bodenmann G. (2009). The role of stress on close relationships and marital satisfaction. Clin. Psychol. Rev. 29, 105–115. 10.1016/j.cpr.2008.10.004 19167139

[B120] Randall A. K. Hilpert P. Jimenez-Arista L. E. Walsh K. J. Bodenmann G. (2016). Dyadic coping in the U.S.: psychometric properties and validity for use of the english version of the dyadic coping inventory. Curr. Psychol. 35, 570–582. 10.1007/s12144-015-9323-0

[B121] Regan T. Lambert S. D. Kelly B. (2013). Uptake and attrition in couple-based interventions for cancer: perspectives from the literature. Psychooncology 22, 2639–2647. 10.1002/pon.3342 23840033

[B122] Regan T. W. Lambert S. D. Girgis A. Kelly B. Kayser K. Turner J. (2012). Do couple-based interventions make a difference for couples affected by cancer? a systematic review. BMC Cancer 12:279. 10.1186/1471-2407-12-279 22769228 PMC3464780

[B123] Regan T. W. Lambert S. D. Kelly B. Falconier M. Kissane D. Levesque J. V. (2015). Couples coping with cancer: exploration of theoretical framewoks from dyadic studies. Psychooncology 24, 1605–1617. 10.1002/pon.3854 26059915

[B124] Revenson T. Lepore S. J. (2012). Coping in Social Context. in Handbook of Health Psychology, 2nd Edn, eds. Baum A. Revenson T. A. Singer J. (New York, NY: Taylor and Francis Group), 193–217.

[B125] Revenson T. A. (1994). Social support and marital coping with chronic illness. Ann. Behav. Med. 16, 122–130.

[B126] Revenson T. A. (2003). Scenes from a marriage: Examining supportand marital coping with chronic illness, in Social Psychological Foundations of Health and Illness, eds. Suls J. Wallston K. (Oxford: Blackwell Publishing), 530–559.

[B127] Revenson T. A. DeLongis A. (2011). Couples coping with Chronic Illness, in The Oxford Handbook of Stress, Health and Coping, ed. Folkman S. (New York, NY: Oxford University Press), 101–123.

[B128] Revenson T. A. Kayser K. Bodenmann G. (2005). Couples Coping With Stress: Emerging Perspectives on Dyadic Coping. Washington, DC: American Psychological Association.

[B129] Revenson T. A. Pranikoff J. R. (2005). A contextual approach to treatment decision making among breast cancer survivors. Health Psychol. 24, S93–S98. 10.1037/0278-6133.24.4.S93 16045426

[B130] Ruddy K. J. Greaney M. L. Sprunck-Harrild K. Meyer M. E. Emmons K. M. Partridge A. H. (2013). Young women with breast cancer: a focus group study of unmet needs. J. Adolesc. Young Adult Oncol. 2, 153–160. 10.1089/jayao.2013.0014 24380034 PMC3869463

[B131] Ruffieux M. Nussbeck F. W. Bodenmann G. (2014). Long-term prediction of relationship satisfaction and stability by stress, coping, communication, and well-being. J. Divorce Remarriage 55, 485–501. 10.1080/10502556.2014.931767

[B132] Saita E. (2009). Psico-Oncologia: Una Prospettiva Relazionale. Milano: Edizioni Unicopli.

[B133] Scabini E. Rossi G. (2012). Family Transitions and Families in Transitions. Milano: VitaandPensiero.

[B134] Schulz U. Schwarzer R. (2004). Partnerschaftliche Bewältigung einer Krebserkrankung [dyadic coping with cancer], in Stress Gemeinsam Bewältigen, eds. Buchwald P. Hobfoll S. E. Schwarzer C. (Göttingen: Hogrefe), 121–138.

[B135] Scott J. L. Halford W. K. Ward B. G. (2004). United we stand? the effects of a couple-coping intervention on adjustment to early stage breast or gynaecological cancer. J. Consult. Clin. Psychol. 72, 1122–1135. 10.1037/0022-006X.72.6.1122 15612858

[B136] Scott J. L. Kayser K. (2009). A review of couple-based interventions for enhancing women's sexual adjustment and body image after cancer. Cancer 15, 48–56. 10.1097/PPO.0b013e31819585df 19197174

[B137] Shahi V. Lapid M. I. Kung S. Atherton P. J. Sloan J. A. Clark M. M.. (2014). Do age and quality of life of patients with cancer influence quality of life of the caregiver? J. Geriatr. Oncol. 5, 331–336. 10.1016/j.jgo.2014.03.003 24726867 PMC4364547

[B138] Sjolander C. Rolander B. Järhult J. Mårtensson J. Ahlstrom G. (2012). Health-related quality of life in family members of patients with an advanced cancer diagnosis: a one-year prospective study. Health Qual. Life Outcomes 10:89. 10.1186/1477-7525-10-89 22846452 PMC3489687

[B139] Skerrett K. Fergus K. (2015). Couple Resilience. New York, NY: Springer.

[B140] So W. K. Choi K. C. Chan C. W. Chair S. Y. (2011). Age-related differences in the quality of life of Chinese women undergoing adjuvant therapy for breast cancer. Res. Gerontol. Nurs. 4, 19–26. 10.3928/19404921-20101201-01 21210577

[B141] Staff H. R. Didymus F. F. Backhouse S. H. (2017): The antecedents outcomes of dyadic coping in close personal relationships: a systematic review narrative synthesis, Anxiety Stress Coping 30, 498–520. 10.1080/10615806.2017.1329931 28513191

[B142] Stava C. J. Lopez A. Vassilopoulou-Sellin R. (2006). Health profiles of younger and older breast cancer survivors. Cancer 107, 1752–1759. 10.1002/cncr.22200 16967441

[B143] Sullivan K. T. Pasch L. A. Johnson M. D. Bradbury T. N. (2010). Social support, problem solving, and the longitudinal course of newlywed marriage. J. Pers. Soc. Psychol. 98, 631–644. 10.1037/a0017578 20307134

[B144] Traa M. J. De Vries J. Bodenmann G. Den Oudsten B. L. (2015). Dyadic coping and relationship functioning in couples coping with cancer: a systematic review. Br. J. Health Psychol. 20, 85–114. 10.1111/bjhp.12094 24628822

[B145] van Steenbergen E. F. Kluwer E. S. Karney B. R. (2011). Workload and the trajectory of marital satisfaction in newlyweds: job satisfaction, gender, and parental status as moderators. J. Fam. Psychol. 25, 345–355. 10.1037/a0023653 21553965

[B146] Vanlemmens L. Christophe V. Fournier E. Dauchy S. Boinon D. Toudic-Emily F. (2012a). The quality of life of young women with nonmetastatic braest cancer and their partners': specific needs require development of scientific questionnaires for each of them. Breast J. 18, 182–184. 10.1111/j.1524-4741.2011.01218.x 22284212

[B147] Vanlemmens L. Fournier E. Boinon D. Machavoine J. L. Christophe V. (2012b). Quality of life of young women with early breast cancer and their partners: specific needs result in the necessity of development of specific questionnaires for the patients and the partner. Bull. Cancer 99, 685–691. 10.1684/bdc.2012.1598 22640925

[B148] Vedes A. Nussbeck F. Bodenmann G. (2015). A life cycle perspective on stress, dyadic coping and couples' well-being, in Paper presented at the 9yh Workshopkongress für Klinische Psychologie und Psychoterapie/33. Symposim der Fachgruppe Klinische Psychologie und Psychotherapie der DGPs (Dresden).

[B149] Vilchinsky N. Liat Haze-Filderman L. H. Morton Leibowitz M. Reges O. Khaskia A. Mosseri M. (2010). Spousal support and cardiac patients' distress: the moderating role of attachment orientation. J. Fam. Psychol. 24, 508–512. 10.1037/a0020009 20731497

[B150] Walsh S. R. Manuel J. C. Avis N. E. (2005). The impact of breast cancer on younger women's relationships with their partner and children. Fam. Syst. Health 23, 80–93. 10.1037/1091-7527.23.1.80

[B151] Webster K. Cella D. Yost K. (2003). The Functional Assessment of Chronic Illness Therapy (FACIT) measurement system: properties, applications, and interpretation. Health Qual. Life Outcomes 1:79. 10.1186/1477-7525-1-79 14678568 PMC317391

[B152] Webster K. Odom L. Peterman A. Lent L. Cella D. (1999). The Functional Assessment of Chronic Illness Therapy (FACIT) measurement system: validation of version 4 of the core questionnaire. Qual. Life Res. 8:604 10.1186/1477-7525-1-79.

[B153] Williamson H. C. Karney B. R. Bradbury T. N. (2013). Financial strain and stressful events predict newlyweds' negative communication independent of relationship satisfaction. J. Fam. Psychol. 27, 65–75. 10.1037/a0031104 23421833 PMC3667200

[B154] Winstead-Fry P. Schultz A. (1997). Psychometric assessment of the Functional Assessment of Cancer Therapy-General (FACT-G) scale in a rural sample. Cancer 79, 2446–2452. 9191537

[B155] Wise M. Schatell D. Klicko K. Burdan A. Showers M. (2010). Successful daily home hemodialysis patient-care partner dyads: benefits outweigh burdens. Hemodial. Int. 14, 278–288. 10.1111/j.1542-4758.2010.00443.x 20491970

[B156] Woszidlo A. Segrin C. (2013). Negative affectivity and educational attainment as predictors of newlyweds' problem solving communication and marital quality. J. Psychol. 147, 49–73. 10.1080/00223980.2012.674069 23472443

[B157] Wunderer E. Schneewind K. A. (2008). The relationship between marital standards, dyadic coping and marital satisfaction. Eur. J. Soc. Psychol. 38, 462–476. 10.1002/ejsp.405

